# A Ferroptosis-Related Genes Model Allows for Prognosis and Treatment Stratification of Clear Cell Renal Cell Carcinoma: A Bioinformatics Analysis and Experimental Verification

**DOI:** 10.3389/fonc.2022.815223

**Published:** 2022-01-27

**Authors:** Jiyue Wu, Zejia Sun, Qing Bi, Wei Wang

**Affiliations:** ^1^Department of Urology, Beijing Chaoyang Hospital, Capital Medical University, Beijing, China; ^2^Institute of Urology, Capital Medical University, Beijing, China

**Keywords:** clear cell renal cell carcinoma, ferroptosis, stratified model, individualized treatment, bioinformatics

## Abstract

**Introduction:**

Clear cell renal cell carcinoma (ccRCC) is a malignant tumor characterized by poor prognosis and difficult treatment. Ferroptosis is a relatively new form of programmed cell death that involved in cancer development and therapy resistance. Studies have shown that targeted ferroptosis may be a novel option for the treatment of ccRCC, but key genes and their roles between ferroptosis and ccRCC are limited so far. This study aims to develop a ccRCC stratified model based on ferroptosis-related genes to provide a reference for the prognosis prediction and the individualized treatment of ccRCC.

**Materials and Methods:**

The mRNAs expression data of ccRCC and FRGs were obtained from TCGA and FerrDb database, respectively. Through multiple analysis, a 4-FRG based prognostic stratified model was constructed and its predictive performance was validated through various methods. Then, a nomogram based on the model was constructed and ccRCC patients stratified by the model were analyzed for tumor microenvironment, immune infiltration, sensitivity for immune checkpoint inhibitors (ICIs)/traditional anti-tumor therapy and tumor mutation burden (TMB). Functional enrichment analysis was performed to explore potential biological pathways. Finally, we verified our model by RT-qPCR, siRNA transfection, scratch assay and CCK-8 assay.

**Results:**

In this study, the stratified model and a model-based nomogram can accurately predict the prognosis of ccRCC patients in TCGA database. The patients stratified by the model showed different tumor microenvironments, immune infiltration, TMB, resistance to traditional and ICIs therapy, and sensitivity to ferroptosis. Functional enrichment analysis suggested several biological pathways related to the process and prognosis of ccRCC. RT-qPCR confirmed the differential expression of ferroptosis-related genes. Scratch assay and CCK-8 assay indicated the promotion effects of CD44 on the proliferation and migration of ccRCC.

**Conclusion:**

In this study, we established a novel ccRCC stratified model based on FRGs, which can accurately predict the prognosis of ccRCC patients and provide a reference for clinical individualized treatment.

## Background

Clear cell renal cell carcinoma (ccRCC) is the most common type of kidney cancer (1). Clinically, the main treatment for ccRCC is surgical resection as it is not sensitive to radiotherapy and chemotherapy (2). Unfortunately, it is estimated that up to 40% of patients will relapse after surgery. Moreover, once ccRCC develops distant metastasis, the long-term survival of patients will be significantly reduced (1). In the past few years, with the development of immunotherapy, immune checkpoint inhibitors (ICIs) targeting programmed death 1/programmed death ligand 1 (PD-1/PD-L1) and cytotoxic T lymphocyte associated antigen 4 (CTLA-4) brought new hope for the treatment of ccRCC (3). However, studies have shown that less than 50% of ccRCC patients can respond to ICIs treatment (4, 5). Given the poor prognosis and difficult treatment of ccRCC, it is necessary to exploit a new stratified model to evaluate the clinical characteristics, treatment sensitivity of ccRCC patients, and to screen candidate groups, so as to guide the clinical individualized treatment of ccRCC.

Ferroptosis is a relatively new iron-dependent programmed cell death characterized by the accumulation of lipid peroxidation and iron in cells (6). It has been found that ferroptosis is involved in the process of many diseases such as nervous system diseases, organ ischemia-reperfusion injury and tumors (7, 8). Besides, recently ferroptosis was found to be involved in the progression and drug resistance of a variety of cancers (9, 10). Induction of ferroptosis can cause tumor cytotoxic lipid peroxidation and mitochondrial dysfunction, thereby inhibiting tumor development (9). Therefore, selective induction of ferroptosis is an effective method to cause tumor cell death, especially for malignant tumors that are resistant to traditional therapies (11, 12). Tang et al. found that cystine deprivation significantly inhibited the survival of VHL-deficient ccRCC (13). Cystine deprivation can lead to the synthesis disorder of reduced glutathione (GSH). The decrease of GSH reduces the resistance of cells to oxidative stress, which causes the accumulation of ROS and leads to ferroptosis (7). Besides, Yang et al. conducted cell experiments on 60 cell lines obtained from 8 tissues and confirmed that compared to other cell lines, RCC cell lines are more sensitive to Erastin-induced ferroptosis (14). Therefore, targeting to induce ferroptosis or block the ferroptosis resistance pathway has great potential in the treatment of ccRCC.

Recently, with the progress of research, many genes have been identified as important regulators or biomarkers of ferroptosis. Wang et al. found that SUV39H1 (one of the methyltransferases) can inhibit ferroptosis to promote tumor progression by regulating the H3K9me3 status of the DPP4 gene promoter region (15). Nuclear receptor coactivator 4 (NCOA4)-mediated ferritinophagy can increase the level of free iron in the cytoplasm to promote ferroptosis (16). CHAC1 gene can be up-regulated in response to erastin-induced ferroptosis and has been suggested as a biomarker for erastin-induced ferroptosis (17). It can be seen that ferroptosis-related genes (FRGs) may be closely related to the process of ccRCC (may promote ccRCC progression or inhibit). However, a lot of details between FRGs and ccRCC patients remain unknown. Most studies focus on a single gene such as SLC7A11 and PRAS40 (18, 19). Therefore, there is an urgent need to develop a ccRCC patient stratified model based on FRGs to measure the prognosis and treatment sensitivity, so as to help timely intervention and individualized treatment.

In this study, we screened out the differentially expressed FRGs (DE-FRGs) between normal and ccRCC specimens. Then, we constructed a prognostic stratified model containing 4 FRGs through univariate Cox regression analysis, Least Absolute Shrinkage and Selection Operator (LASSO) regression analysis and multivariate Cox regression analysis. The stratified model and a model-based nomogram can accurately predict the prognosis of ccRCC patients in TCGA database. This model provides a theoretical basis for patients grouping among tumor microenvironment, immune infiltration, tumor mutation burden (TMB), resistance to therapy and sensitivity to ferroptosis, which facilitates individualized treatment. Finally, we also explored the potential biological pathways.

## Materials and Methods

### Screening of the DE-FRGs

First, the mRNAs sequencing data derived from 539 ccRCC specimens and 72 adjacent nontumor renal specimens, and the corresponding clinical information of ccRCC patients were downloaded from The Cancer Genome Atlas (TCGA) database (http://tcga.cancer.gov). A total of 530 ccRCC patients with complete clinical information were included in this study and **Table S1** showed the characteristics of all patients. Then, we obtained FRGs from the FerrDb online database (http://www.zhounan.org/ferrdb/). By integrating the above-mentioned mRNAs with FRGs, the FRGs expressed in ccRCC were obtained. Finally, we identified the DE-FRGs between ccRCC and normal specimens using the “edgeR” package in the R software (20). The threshold was set to |log_2_FC|>1 and the P value <0.05. Log2 transformation was performed on the expression levels of DE-FRGs for subsequent analysis.

### Construction of the Prognostic Stratified Model

We randomly divided the patients into the training set and the testing set according to the ratio of 7:3. The training set was used for constructing the prognostic stratified model of ccRCC patients, while the testing set was used for validating the model. First, using a univariable Cox regression analysis (HR ≠ 1, p<0.05) to screen out prognosis-related FRGs (PR-FRGs) in the training set and intersected these PR-FRGs with DE-FRGs to get overlapping candidate FRGs (OC-FRGs). Then, a LASSO regression analysis with 1000-folds cross-validation was conducted on the OC-FRGs for sub-selection using the “glmnet” package in the R software (21). Finally, a multivariable Cox regression analysis was performed on the sub-selected FRGs to determine the optimal PR-FRGs. Linearly combining the expression level and regression coefficient obtained in the multivariable Cox regression analysis of each optimal PR-FRG to obtain the prognostic stratified model:


$$Risk\;score=\sum_{i=1}^{n}\left({\text{coef}}_{i}\times {\text{Exp}}_{i}\right)$$

The *coef* represents the regression coefficient of optimal PR-FRG in patient *i*, while the *Exp* is its expression level.

### Assessment of the Prognostic Stratification Model

We stratified ccRCC patients into high-and low-risk groups based on the median risk score in the training set. Survival between the two groups was compared through the Kaplan–Meier (K–M) survival analysis and the predictive performance of our model was evaluated by the time‐dependent receiver operating characteristic (ROC) curve (22). In addition, we also performed the K-M survival analysis in different clinical subgroups (including genders, ages, histologic grade, and pathologic stage). Finally, the predictive performance and applicability of our model were also validated in the testing set and the entire set.

### Independent Prognosis Analysis and Construction of the Nomogram

To evaluate whether the stratified model is independent of other clinical parameters such as age, gender, histologic grade and pathologic stage. The survival of ccRCC patients was set as the dependent variable, while the risk score and other clinical parameters as independent variables to perform a univariable and a multivariable Cox regression analysis.

To build a more accurate and reliable clinical prognostic tool, a nomogram was constructed by integrating the results of the independent prognostic analysis *via* the “rms” package of the R software (23). The predictive performance of our nomogram was evaluated by calibration plots. The Y-axis represents the actual survival while the X-axis represents the predicted survival and the 45° dotted line represents the best outcome. Besides, the K-M analysis and the ROC curve were also performed to measure the nomogram.

### Calculation of Tumor Microenvironment and Immune Infiltration

We performed ESTIMATE algorithm on the downloaded mRNAs expression data to evaluate the tumor microenvironment of each ccRCC patient using the “estimate” package in the R software (24). ESTIMATE is a complex algorithm that estimates the infiltration of tumor cells and other cells based on the transcription profile of cancer tissue.

CIBERSORT algorithm with 1000 simulations was used to analyze the infiltrating immune cells of each ccRCC sample. This algorithm was used to analyze the 22 distinct leukocyte subsets in the cancer tissue based on the transcriptome data (25).

### Analysis of TMB

TMB represents the number of mutations per million bases in tumor tissue, including genetic coding errors, base substitutions, base insertions and base deletions (26). In theory, tumor tissues with higher TMB can be more easily recognized by the immune system, so immunotherapy against them is more effective. To this end, we calculated the TMB score of each patient based on the somatic mutation data of ccRCC patients obtained from the TCGA database. Besides, we also evaluated the correlation between the TMB score and the risk score based on the stratified model.

### Analysis of Immune Checkpoint and Drug Sensitivity

ICI is an effective treatment strategy against a variety of tumors. In this study, we compared whether the expression levels of immune checkpoint molecules CTLA-4 and PD-L1 between the two groups were different, thereby assessing whether the stratified model could screen out ccRCC patients sensitive to ICI.

Besides, we also obtained the IC50 of anti-tumor drugs and the mRNAs expression profile of ccRCC cell lines from the GDSC database (27). Through Pearson correlation analysis, anti-tumor drugs whose IC50 (P<0.05 and |R|>0.3) were significantly correlated with the risk score were selected.

### Functional Enrichment Analysis

Gene ontology (GO) annotation and Kyoto Encyclopedia of Genes and Genomes (KEGG) pathway enrichment analysis were conducted on the DE-FRGs identified between tumor and normal specimens with the “clusterProfiler” and “enrichplot” packages in the R software (28). P<0.05 was considered as statistically significant.

For the optimal PR-FRGs finally screened, Gene Set Enrichment Analysis (GSEA) analysis was performed to detect potential biological processes with GSEA software (v4.1.0). The reference set was the annotated gene set file (c2.cp.kegg.v7.2.symbols.gmt) obtained from the molecular marker technology database (MSigDB) (29). The number of permutations was 1000 and an enriched gene set was got when P<0.05 and FDR<0.05.

### Cell Cultures, Total RNA Isolation and RT-qPCR

The human renal cortex proximal tubule epithelial cells (HK-2 cells) were cultured in DMEM/F12 medium (Gibco, USA), the human primary clear cell adenocarcinoma cells (7860 cells) and the human skin metastasis-derived clear cell renal cell carcinoma cells (Caki-1 cells) were maintained in RPMI 1640 medium (Gibco, USA) and McCoy’s 5A Medium (iCell, China), respectively. All mediums were supplemented with 10% fetal bovine serum (Gibco, USA), 100 U/mL of penicillin and 100 U/mL of streptomycin (Gibco, USA). The conditions of cell cultures were 37°C and 5% CO2.

Using the FastPure Cell/Tissue Total RNA Isolation Kit (Vazyme, China) to extract the total RNA following the manufacturer’s instructions. Then, the HiScript III RT SuperMix for qPCR (+gDNA wiper) (Vazyme, China) was used to perform the reverse transcription reactions and the AceQ Universal SYBR qPCR Master Mix (Vazyme, China) was used to complete qPCR.

The comparative Ct method was used to analyze the relative expression levels of four FRGs: 2−ΔΔCt (ΔΔCt = (ΔCt of FRG) − (ΔCt of GAPDH). GAPDH was used as the internal normalized reference to FRGs. The specific primer sequences used were listed in **Table 1**.

**Table 1:** Primer sequences of related genes for RT-qPCR.

Gene		Sequence (5’- 3’)
CD44	F*	GGACAAGTTTTGGTGGCAC
	R^#^	CCGTCCGAGAGATGCTGTA
BID	F	TCCTCCAAAGCTGTTCTGAC
	R	TTATTGTCTGACGCCCCTG
TRIB3	F	TCTGGTCCTGCGTGATCTC
	R	GTATCTCAGGTCCCACGTAGG
TAZ	F	ATCACCACATGGCAAGACC
	R	GTTCTGCTGGCTCAGGGTA
GAPDH	F	GCCTTCCGTGTCCCCACTGC
	R	GGCTGGTGGTCCAGGGGTCT

*F, forward; ^#^R, reverse.

### RNA Interference and RT-qPCR

The 7860 and Caki-1 cells were respectively seeded into plates at an appropriate density. According to the manufacturer’s protocols, small interfering RNA (siRNA, 50-100 nmol/L) and lipofectamine RNAiMAX transfection reagent (Invitrogen, Carlsbad, CA, USA) were used for transfection. Subsequently, RT-qPCR was performed to detect the efficiency of gene knockdown.

### Cell Viability and Scratch Assay

After the 7860 and Caki-1 cells were transfected for 48 hours, 100 μL of Eppen-dorf Tip was used to scratch the cell plate and the cells were washed 2-3 times to remove cellular debris. Observe the changes of cells in each group at 0h, 6h, 12h, and 24h with an inverted microscope.

The 7860 and Caki-1 cells were divided into groups of Caki-1+Si CD44 NC, Caki-1+Si CD44, 786O+Si CD44 NC, 786O+Si CD44. After 48 hours of transfection, the cells were digested with trypsin and were seeded in a 96-well plate at 3×10^3^ cells/well. After 6, 12, 24, and 48 hours, 10μL of Cell Counting Kit-8 (CCK8) (DoJinDo, Japan) was added to each well and incubated at 37°C for two hours. Then using a microplate reader (Thermo, USA) to measure the absorbance at 450nm.

### Statistical Analysis

All statistical analyses in this study were conducted on R software (version 3.6.3) and statistical tests were all two-sided. P<0.05 was considered as statistically significant. Statistical significance between two groups was evaluated by Student’s t test. All experiments were repeated at least three times.

## Results

### Screening of the DE-FRGs

**Figure 1:**
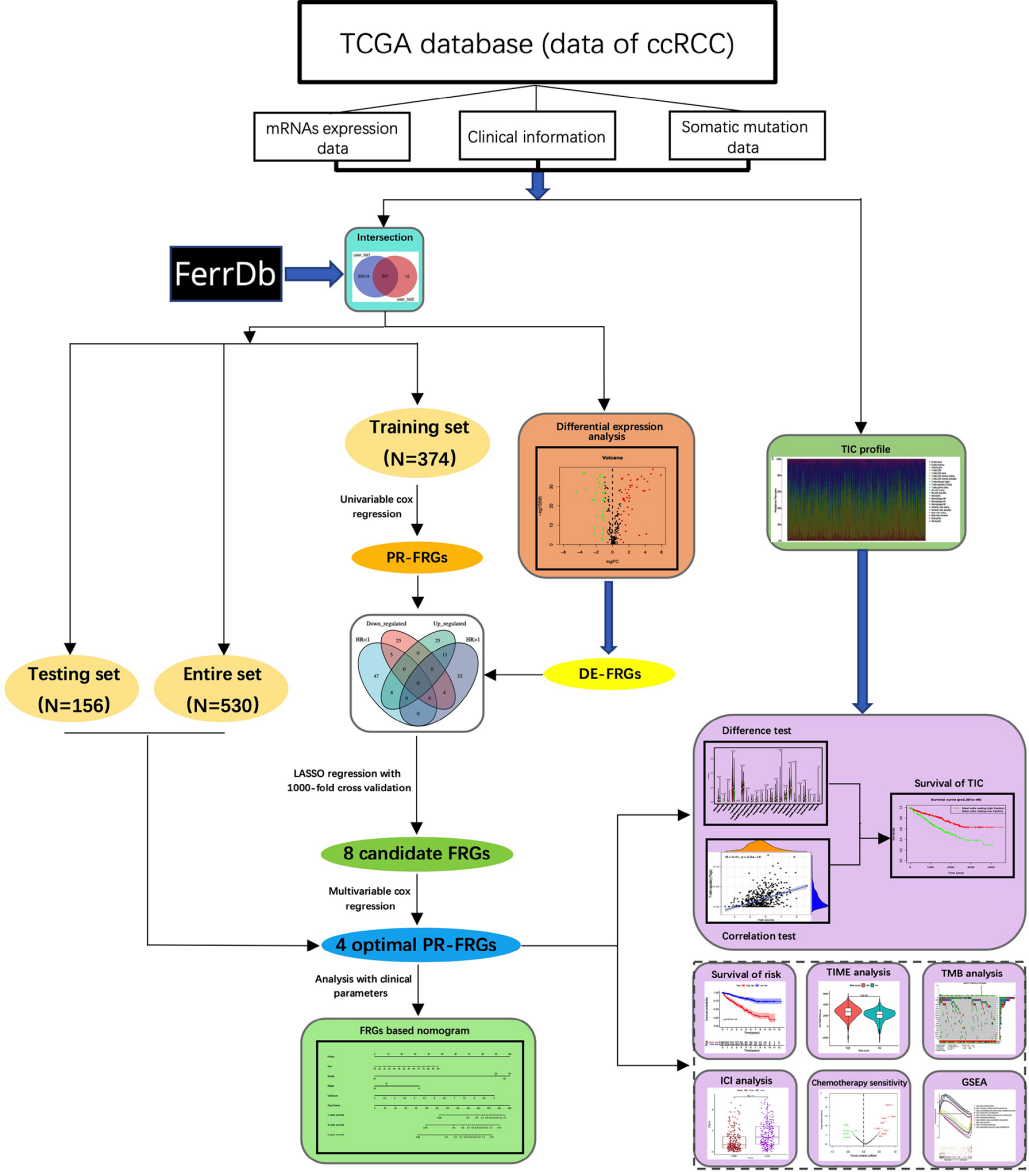
The flowchart of this study. In this study, we obtained the mRNAs sequencing data of ccRCC from the TCGA database and integrated it with the FRGs in the FerrDb database. Then, we screened the optimal FRGs through univariable cox regression, LASSO regression and multivariable cox regression, and finally built a model based on four FRGs. Next, we stratified ccRCC patients according to our model and explored the differences between the two groups of patients in terms of clinical prognosis, TIME, TMB, treatment, and potential biological processes. CcRCC, clear cell renal cell carcinoma; FRGs, ferroptosis-related-genes; LASSO, Least Absolute Shrinkage and Selection Operator; DE-FRGs, differentially expressed FRGs; PR-FRGs, prognosis-related FRGs; TIC, tumor immune cell; TIME, tumor immune microenvironment; TMB, tumor mutation burden; ICIs, immune checkpoint inhibitors; GSEA, Gene Set Enrichment Analysis.

**Figure 2:**
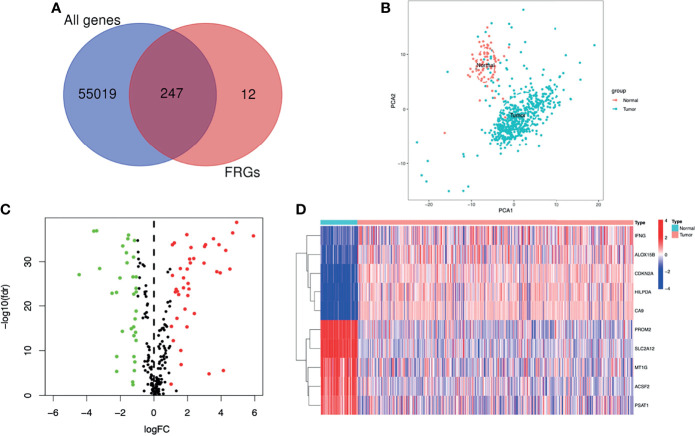
Screening of the DE-FRGs. **(A)** The FRGs expressed between ccRCC specimens and normal specimens. **(B)** A PCA plot of the expression profile of FRGs between tumor and normal specimens. **(C)** Volcano plot of DE-FRGs between tumor and normal specimens. The green dots represent DE-FRGs with log2FC < -1 and the red dots represent DE-FRGs with Log2FC > 1. **(D)** Heatmap of the top 5 up- and down-regulated FRGs. DE-FRGs, differentially expressed FRGs; PCA, Principal component analysis.

The flowchart of this study was shown in **Figure 1**. A total of 55266 mRNAs were obtained from the sequencing data of ccRCC patients in the TCGA database and 259 FRGs were got from the FerrDb online database. After intersecting the above-mentioned mRNAs and FRGs, we obtained 247 FRGs expressed in ccRCC (**Figure 2A**). Principal component analysis (PCA) indicated that the ccRCC specimens and normal specimens had significantly different expression profiles of FRGs (**Figure 2B**). Next, we identified 76 DE-FRGs between the two specimens, including 42 up-regulated FRGs and 34 down-regulated FRGs (**Figure 2C**). The Log_2_FC of these DE-FRGs with their p values were listed in **Table S2**. **Figure 2D** showed the heatmap of the top 5 up-and down-regulated FRGs. The top 5 up-regulated FRGs in ccRCC were IFNG, ALOX15B, CDKN2A, HILPDA and CA9, while the top 5 down-regulated FRGs in ccRCC were PROM2, SLC2A12, MT1G, ACSF2 and PSAT1.

### Construction of the Prognostic Stratified Model

**Figure 3:**
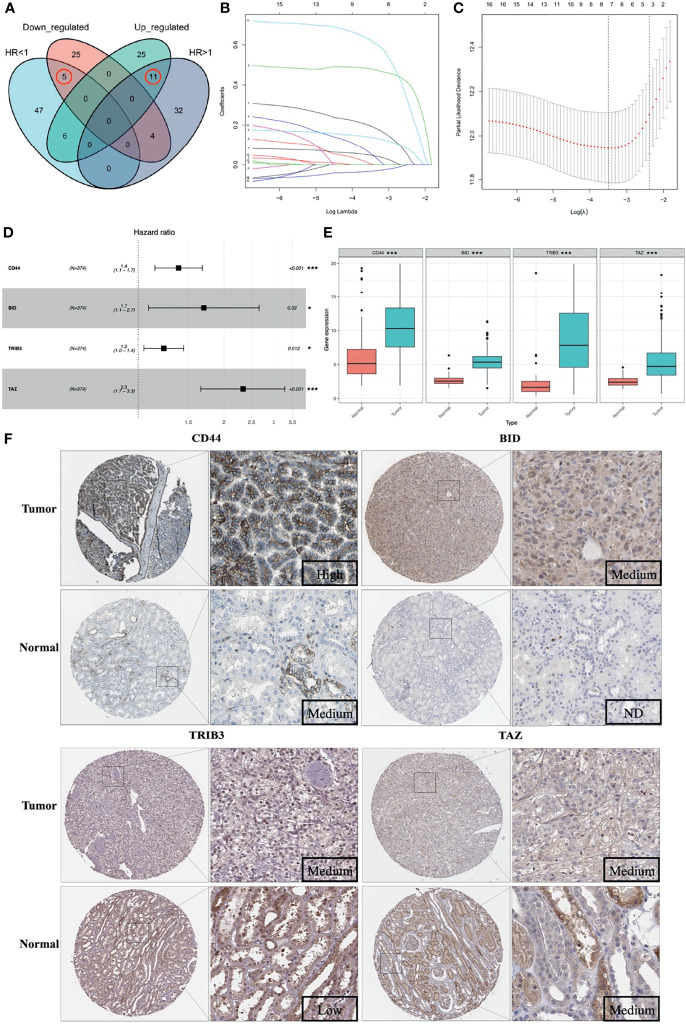
Construction of the prognostic stratified model. **(A)** Venn diagram of 16 OC-FRGs; **(B)** LASSO coefficients profiles of 16 OC-FRGs. **(C)** The 8 candidate FRGs obtained by LASSO regression with 1000-fold cross-validation to identify the optimal lambda value that came from the minimum partial likelihood deviance. **(D)** 4 optimal PR-FRGs got by multivariable Cox regression analysis. **(E)** Expression pattern of the 4 optimal PR-FRGs between tumor and normal specimens. **(F)** Images of immunohistochemistry staining of the 4 optimal PR-FRGs in tumor and normal specimens from the HPA database. OC-FRGs, overlapping candidate FRGs; LASSO, least absolute shrinkage and selection operator; PR-FRGs, prognosis-related FRGs. ND, not detected. * means p < 0.05 and *** means p < 0.001.

**Figure 4:**
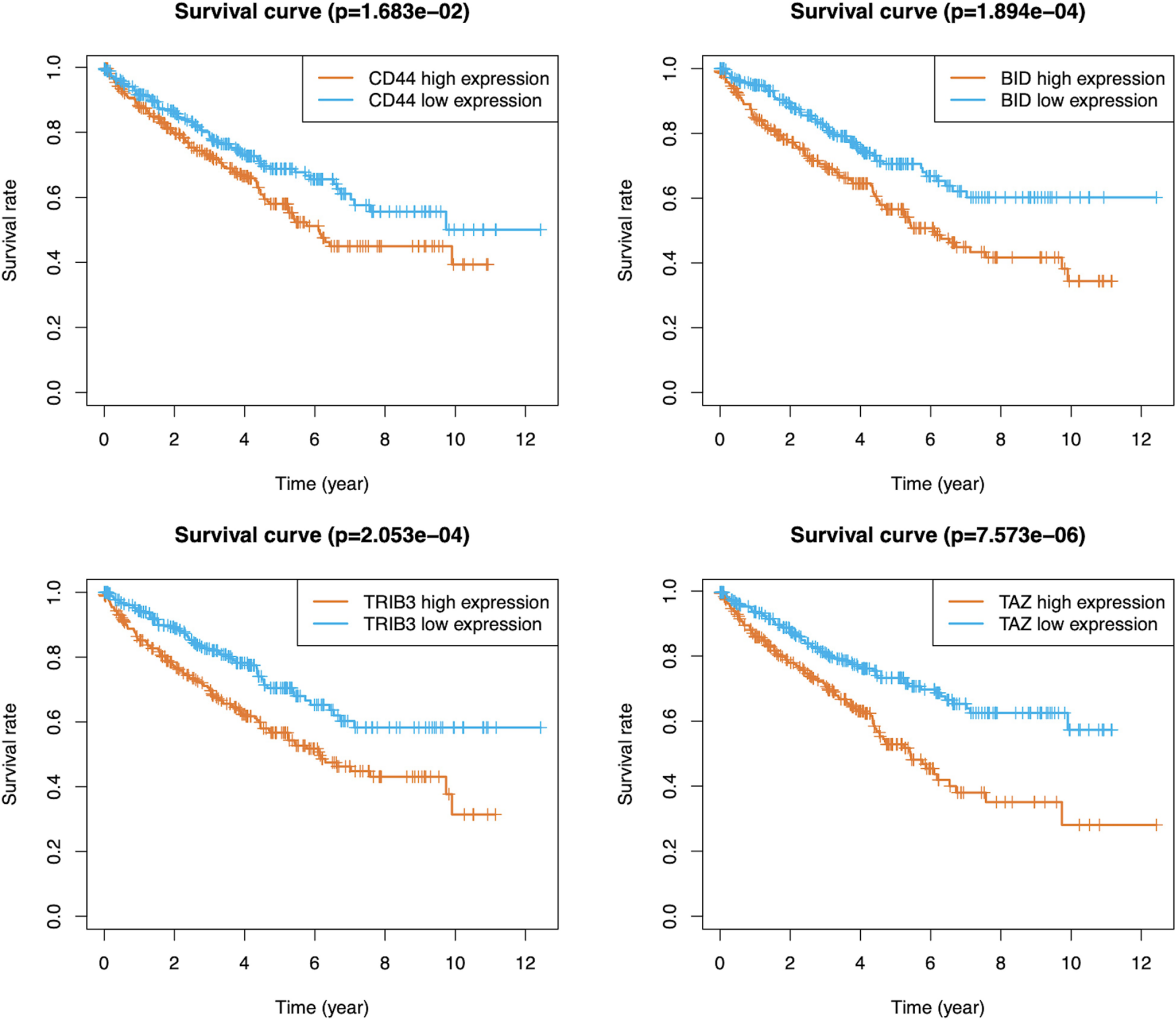
K-M survival analysis of CD44, BID, TRIB3 and TAZ in the TCGA database. K-M, Kaplan–Meier.

A total of 105 PR-FRGs were identified based on our criteria, of which 47 with HR>1 and 58 with HR<1. By intersecting 47 PR- FRGs (HR>1) with 42 DE-FRGs (log_2_FC>1) and 58 PR-FRGs (HR<1) with 34 DE-FRGs (log_2_FC<1) respectively, we obtained 16 OC-FRGs (**Table S4**, **Figure 3A**). Through a LASSO regression with 1000-folds cross-validation to identify the optimal lambda value that came from the minimum partial likelihood deviance, we got 8 candidate FRGs (CD44, BID, DRD4, TRIB3, CDKN2A, SLC7A11, TAZ and MIOX) that were significantly associated with the prognosis of ccRCC (**Figures 3B, C****)**. Subsequently, a multivariable Cox regression analysis was applied to the 8 candidate FRGs and finally 4 optimal PR-FRGs (CD44, BID, TRIB3 and TAZ) were identified (**Figure 3D**). **Figure 3E** presented the expression levels of the optimal PR-FRGs between ccRCC specimens and normal specimens. As we can see, the expression levels of the four FRGs (CD44, BID, TRIB3 and TAZ) in ccRCC specimens were significantly higher than those in normal specimens. Besides, we further evaluated the protein expression of four FRGs in the Human Protein Atlas (HPA) database (**Figure 3F**). As shown in the figure, the protein expression levels of CD44, BID, and TRIB3 in tumor tissues were significantly higher than those in normal tissues. However, it was worth to note that the protein expression level of TAZ showed no difference. The prognostic power of each optimal PR-FRGs for ccRCC patients was shown in **Figure 4**. Furthermore, by assembling the expression level and the corresponding regression coefficients of each optimal PR-FRG, we constructed a prognostic stratified model and the formula was as follows: Risk score=Exp_(CD44)_*0.32649243+Exp_(BID)_*0.52992859+Exp_(TRIB3)_*0.20647281+Exp_(TAZ)_*0.8476897, and calculate the risk score of all ccRCC patients.

### Evaluation of the Prognostic Stratified Model

**Figure 5:**
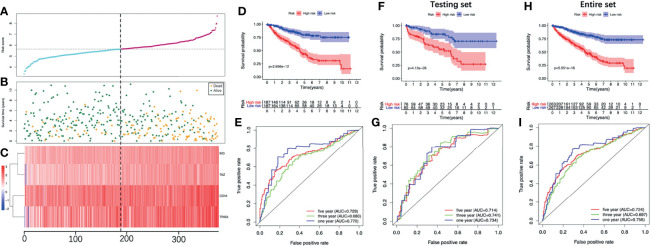
Evaluate the prognostic analysis of the four-FRG model in the training set **(A-E)** and validate in the testing set **(F, G)** and the entire set **(H, I)**. **(A)** The curve of risk score. **(B)** Survival status of the ccRCC patients. **(C)** Heatmap of the expression levels of the four optimal PR-FRGs. The dotted line represented the median risk score and stratified the ccRCC patients into low-risk and high-risk group. **(D)** K-M survival analysis of the model. **(E)** Time-dependent ROC analysis of the model. **(F)** K-M survival analysis in the testing set. **(G)** Time-dependent ROC analysis in the testing set. **(H)** K-M survival analysis in the entire set. **(I)** Time-dependent ROC analysis in the entire set. CcRCC, clear cell renal cell carcinoma; PR-FRGs, prognosis-related FRGs; K-M, Kaplan–Meier; ROC, receiver operating characteristic.

**Figure 6:**
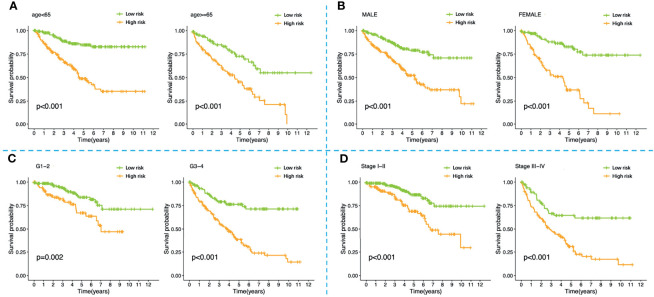
K-M survival analysis of high-risk and low-risk patients stratified by the four-FRG model in different clinical subgroups including **(A)** younger than 65 years old, older than 65 years old; **(B)** male, female; **(C)** grade1-2, grade3-4; **(D)** stage I-II, stage III-IV. K-M, Kaplan–Meier.

We stratified patients of the training set into two groups according to the median risk score (5.645) (**Figure 5A**). The survival status of all patients in the training set was shown in **Figure 5B**, as we can see, the number of deaths was more in the high-risk group. **Figure 5C** showed the heatmap of the four optimal PR-FRGs. The K-M survival analysis showed that the long-term survival of the high-risk group was significantly shorter, which suggested that high-risk group may have a poor prognosis (**Figure 5D**). The ROC curve presented the reliable prognostic accuracy of our model with the area under the curve (AUC) was 0.770 at 1 year, 0.680 at 3 years, and 0.729 at 5 years (**Figure 5E**). Besides, the K-M survival analysis in different clinical subgroups also showed that the high-risk group held worse survival than the low-risk group (**Figure 6**). **Supplementary Figure 1** showed the risk scores of patients in different clinical subgroups and **Supplementary Figure 2** showed the PR-FRGs expression between the two groups.

To validate the model we constructed, we further applied the model (cut-off=5.645) in the testing set and the entire set. Consistent with the training set, the K-M survival of both sets showed that the prognosis of the high-risk group was significantly worse (**Figures 5F, G****)**. The AUC of the testing set was 0.734 at 1 year, 0.741 at 3 years and 0.714 at 5 years; while the AUC of the entire set was 0.756 at 1 year, 0.697 at 3 years and 0.724 at 5 years (**Figures 5H, I****)**. These all indicated that the prognostic performance of our model was satisfactory.

### Establishment of the Nomogram

**Figure 7:**
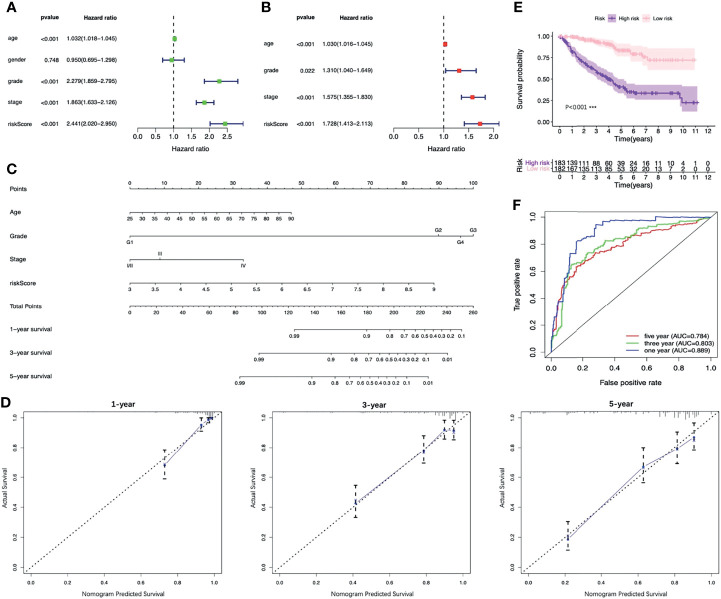
Identifying the independent prognostic factors of ccRCC patients and construction of the nomogram. **(A)** Forrest plot of univariable Cox regression analysis in ccRCC. **(B)** Forrest plot of multivariable Cox regression analysis in ccRCC. **(C)** Nomogram integrated risk score, age, histologic grade and pathologic stage. **(D)** Calibration plots of the nomogram at 1, 3 and 5 years. **(E)** K-M analysis of the nomogram. **(F)** ROC curves of the nomogram. CcRCC, clear cell renal cell carcinoma; K-M, Kaplan–Meier; ROC, receiver operating characteristic.

The results of the univariable analysis showed that the age of patients, the histologic grade, the pathologic stage and the risk score based on the four-FRG model were all negatively correlated with the prognosis of ccRCC patients. The prognosis of older patients with the higher histological grade, the later pathological stage and the higher risk score seem to be worse. In contrast, the gender of the patients was not associated with the prognosis (**Figure 7A**). The risk score and other clinical parameters (including ages of patients, histologic grades, and pathologic stages) were identified as the independent prognostic factors for ccRCC through multivariable analysis (**Figure 7B**).

**Figure 8:**
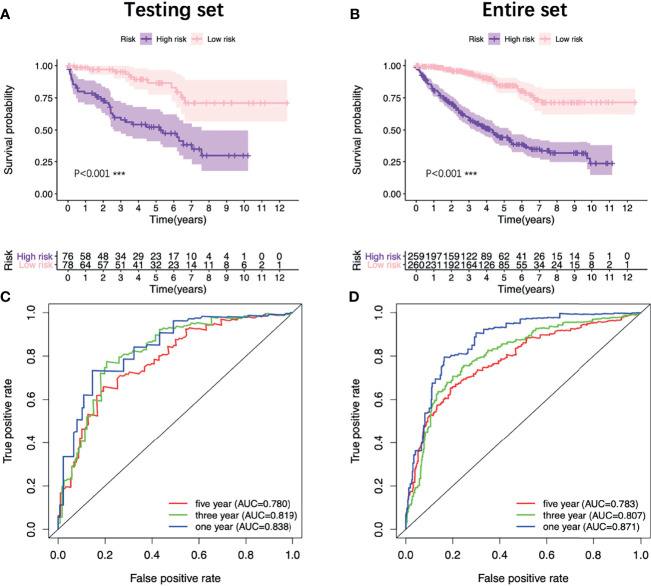
Validation of the nomogram. K-M survival analysis of the nomogram in the testing set **(A)** and the entire set **(B)**. ROC curves of the nomogram in the testing set **(C)** and the entire set **(D)**. K-M, Kaplan–Meier; ROC, receiver operating characteristic.

In order to provide clinicians with a more accurate and reliable prognostic tool, we established a nomogram by integrating the risk score and other three clinical parameters (**Figure 7C**). Calibration plots revealed good agreements between the predicted survival and the actual survival at 1, 3, and 5 years (**Figure 7D**). In addition, the K-M survival analysis and the ROC curve both presented a better prognostic performance of the nomogram in the training set, the testing set and the entire set (**Figures 7E, F**, **8**).

### The Tumor Microenvironment and Immune Infiltration

**Figure 9:**
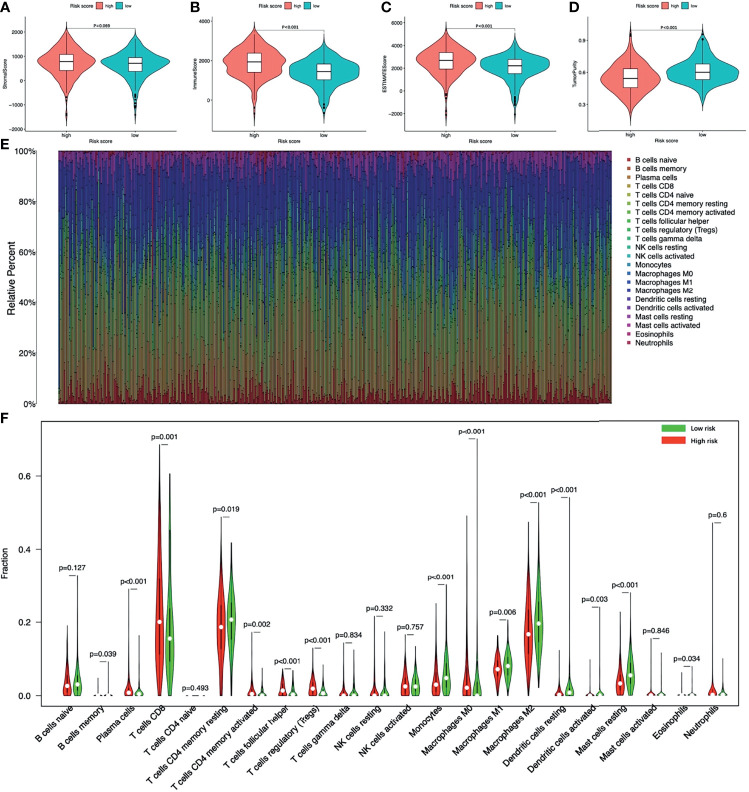
The tumor microenvironment and immune infiltration of ccRCC. The tumor microenvironment between high-risk and low-risk groups **(A)** Stromal score; **(B)** Immune score; **(C)** ESTIMATE score; **(D)** Tumor purity. **(E)** The landscape of immune cell infiltration of all ccRCC patients. **(F)** The infiltrating levels of 22 immune cell types between high-risk and low-risk groups. ccRCC, clear cell renal cell carcinoma.

The tumor microenvironment analysis showed that there was no statistical difference of the Stromal score between the two groups, but the high-risk group had a trend of higher Stromal score (**Figure 9A**). The Immune score and the ESTIMATE score of the high-risk group were significantly higher, while the Tumor purity was opposite (**Figures 9B–D**).

**Figure 10:**
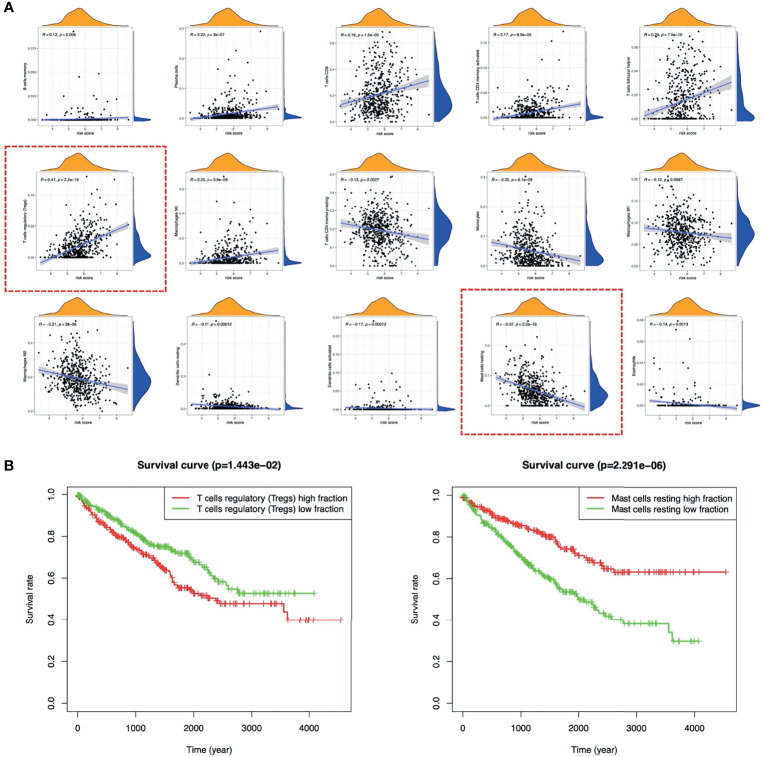
Relationship between TICs and risk score. **(A)** The correlations between the TICs and risk score. **(B)** K-M survival curves of Tregs and Mast cells resting in ccRCC. TICs, tumor immune cells; K-M, Kaplan–Meier; ccRCC, clear cell renal cell carcinoma.

**Figure 9E** showed the landscape of immune cell infiltration in tumor tissues of all ccRCC patients. The abundance of T cells CD4 memory activated, T cells CD8, T cells follicular helper, Tregs, Plasma cells, Macrophages M0 in the high-risk group was significantly higher. While the abundance of T cells CD4 memory, B cells memory, Macrophages M1, Macrophages M2, Monocytes, Dendritic cells resting, Dendritic cells activated, Mast cells resting, Eosinophils was opposite (**Figure 9F**). Among immune cells with different infiltration abundances between the two groups, the abundance of the Mast cells resting and the Tregs were significantly correlated with the risk score (**Figure 10**). The prognostic analysis showed that the abundance of Tregs was negatively correlated with the survival of ccRCC, while the abundance of Mast cells resting was positively correlated with the survival (**Figure 10B**). These results all indicated that the tumor microenvironment and immune infiltration between the two groups were significantly different.

### Tumor Mutation Burden

**Figure 11:**
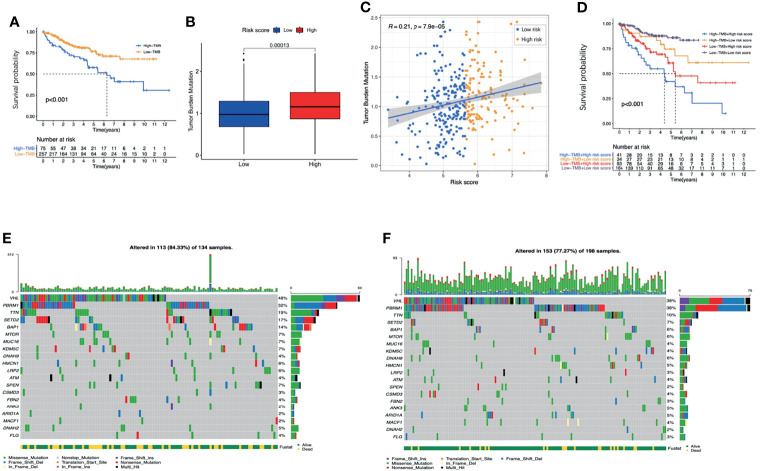
The analysis of TMB. **(A)** K-M survival analysis of the TMB score. **(B)** The TMB scores between high-risk and low-risk groups. **(C)** The correlations between the TMB score and risk score. **(D)** K-M survival analysis of the risk score combined with the TMB score. The waterfall graphs of most frequently mutated genes in high-risk group **(E)** and in low-risk group **(F)**. TMB, tumor mutation burden; K-M, Kaplan–Meier.

By assessing the somatic mutation data of ccRCC patients (n=336), we found that the prognosis of patients with higher TMB was worse than patients with lower TMB (**Figure 11A**). Compared with low-risk patients, high-risk patients had higher TMB scores (**Figure 11B**). Correlation analysis indicated that the TMB score was positively correlated with the risk score (**Figure 11C**, R=0.21, p<0.001). It is worth noting that combining the TMB score with the risk score seemed to have a better prognostic capability for ccRCC patients. Patients with low TMB scores and low risk-scores had the best prognosis, while patients with high TMB scores and high risk-scores had the worst prognosis (**Figure 11D**).

The waterfall graphs further showed the difference of most frequently mutated genes between the two groups (**Figures 11E, F****)**. As we can see, the percentages of the mutations of VHL, TTN, SETD2 and BAP1 in the high-risk group were significantly higher (P<0.05). In conclusion, the TMB between the two groups was significantly different.

### Immune Checkpoint and Drug Sensitivity

**Figure 12:**
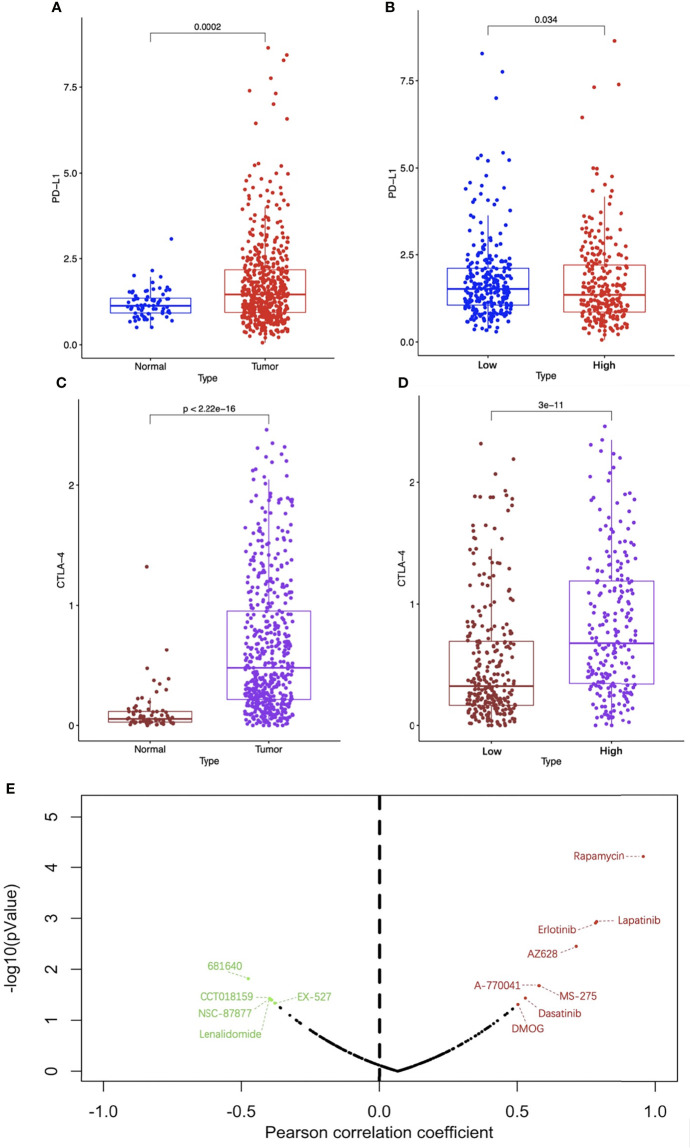
Analysis of immune checkpoint and drug sensitivity. The expression levels of PD-L1 **(A)** and CTLA-4 **(C)** between ccRCC and normal specimens. The expression levels of PD-L1 **(B)** and CTLA-4 **(D)** between high-risk and low-risk groups. **(E)** The correlation between traditional anti-tumor drug sensitivity and risk score, the red dots represent drugs whose IC50 were positively correlated with the risk score with p < 0.05 and the green dots represent drugs whose IC50 were negatively correlated with the risk score with p < 0.05. PD-1, programmed death ligand 1; CTLA-4, cytotoxic T lymphocyte associated antigen 4; CcRCC, clear cell renal cell carcinoma.

ICIs are an emerging effective treatment strategy targeting a variety of tumors, but studies have shown that different patients have significantly different responses to them. Screening out patients who are sensitive to ICIs will contribute to precise and efficient treatment. As shown in **Figures 12A, C**, compared with normal renal specimens, the expression levels of PD-L1 and CTLA-4 were significantly higher in ccRCC specimens. As for high-risk ccRCC patients, the PD-L1 expression was significantly lower than that of low-risk patients, while the CTLA-4 expression was significantly higher (**Figures 12B, D****)**. To explore the sensitivity of ccRCC patients to traditional anti-tumor drugs, we analyzed the IC50 of anti-tumor drugs, the risk score based on the four-FRG model, and the gene expression data of ccRCC cell lines obtained from GDSC database. Pearson correlation analysis showed that the IC50 of eight drugs (including Rapamycin, Lapatinib, Erlotinib, AZ628, A-770041, MS-275, Dasatinib and DMOG) were positively correlated with the risk score, while the IC50 of five drugs (including 681640, CCT018159, NSC-87887, Lenalidomide, and EX-527) were negatively correlated with the risk score (**Figure 12E**).

### Functional Enrichment Analysis

**Figure 13:**
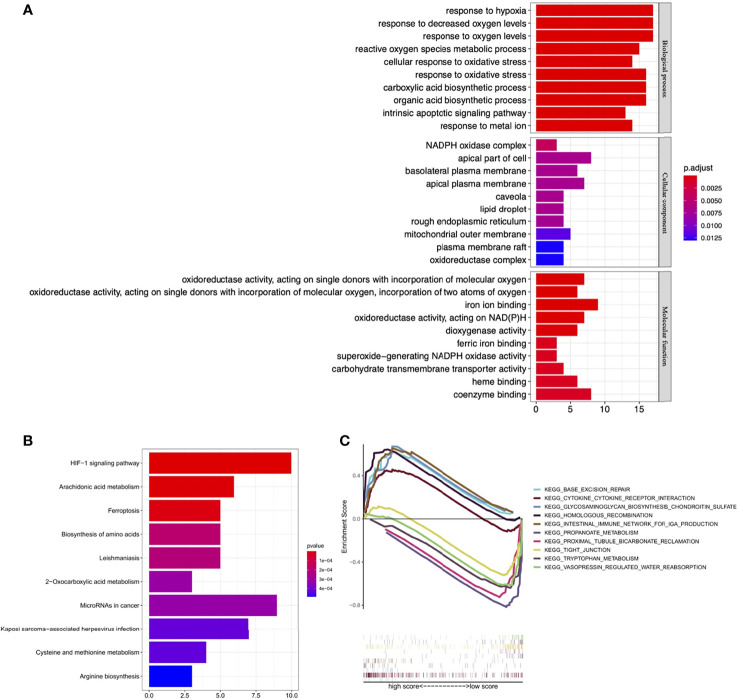
Functional enrichment analysis. **(A)** GO enrichment analysis of the DE-FRGs. **(B)** KEGG enrichment analysis of the DE-FRGs. **(C)** GSEA plot for significant enriched biological processes associated with the four optimal PR-FRGs. GO, gene ontology; KEGG, Kyoto Encyclopedia of Genes and Genomes; GSEA, Gene Set Enrichment Analysis; DE-FRGs, differentially expressed FRGs; PR-FRGs, prognosis-related FRGs.

BP GO analysis showed that DE-FRGs were mainly enriched in the response to hypoxia, response to oxygen levels, reactive oxygen species metabolic process, cellular response to oxidative stress, carboxylic acid biosynthetic process. For CC analysis, DE-FRGs were significantly enriched in NADPH oxidase complex, apical part of cell and basolateral plasma membrane. The MF analysis for DE-FRGs included oxidoreductase activity, iron ion binding and dioxygenase activity (**Figure 13A**). KEGG pathway enrichment analysis showed that these FRGs were mainly enriched in the HIF-1 signaling pathway, Arachidonic acid metabolism, Ferroptosis and Biosynthesis of amino acids (**Figure 13B**).

The results of GSEA analysis showed that Base excision repair, Cytokine-cytokine receptor interaction and Glycosaminoglycan biosynthesis chondroitin sulfate were enriched in high-risk patients, while Propanoate metabolism, Proximal tubule bicarbonate reclamation and Tight junction were mainly enriched in low-risk patients (**Figure 13C**). These results set the foundation for further exploring the potential molecular mechanisms of ccRCC.

### Experimental Verification

**Figure 14:**
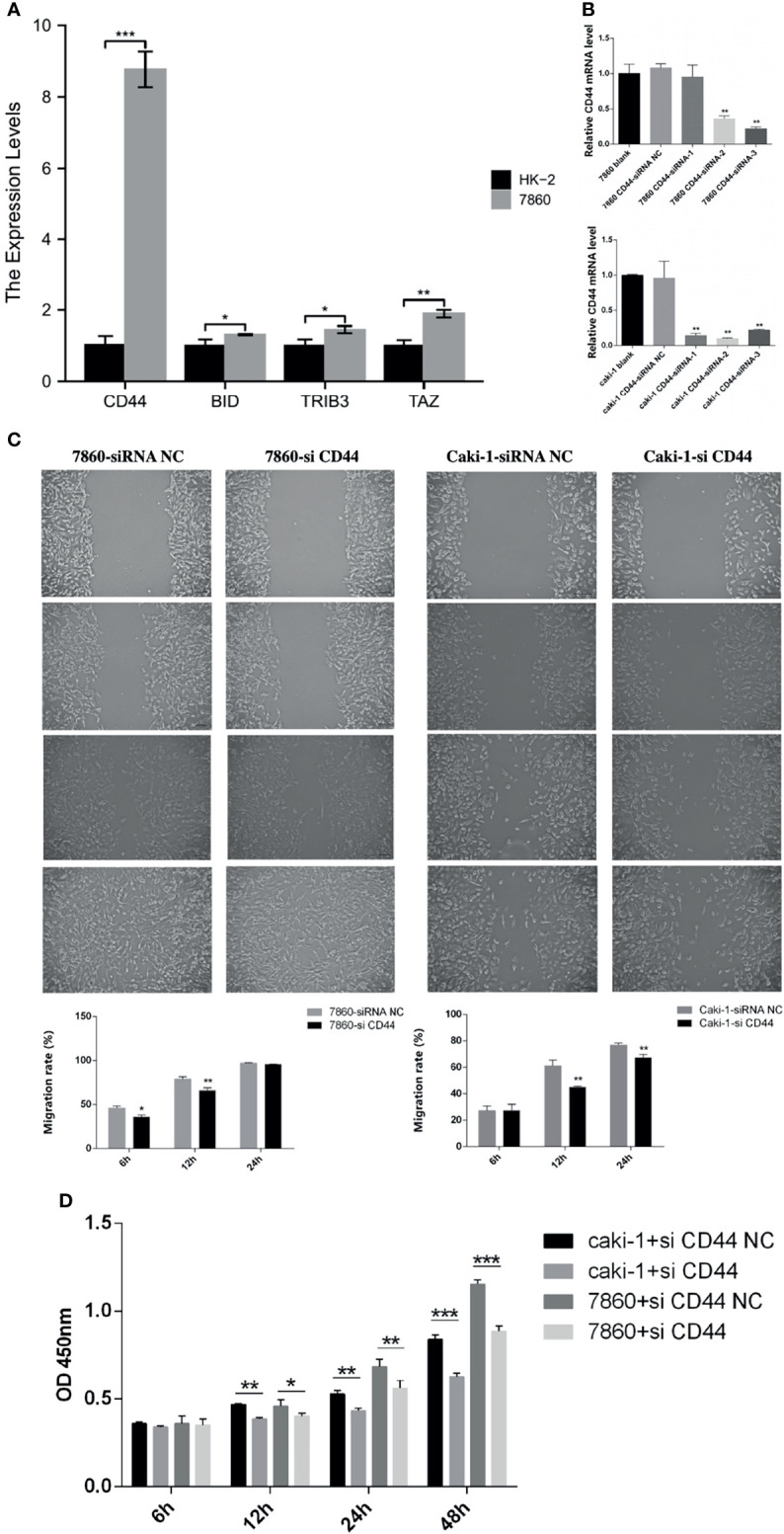
Experimental verification processes. **(A)** The histogram showed the relative expression levels of the 4 FRGs evaluated by RT-qPCR in the HK-2 cells and the 7860 cells. **(B)** Using RT-qPCR to evaluate the efficiency of gene knockdown by siRNA in the 7860 cells and the Caki-1 cells. **(C)** Scratch assay results showed the migration rate of the siRNA-CD44 cells and the siRNA-NC cells. **(D)** CCK-8 assay results showed the relative proliferation the siRNA-CD44 cells and the siRNA-NC cells. All experiments were repeated at least three times, and the data were shown as means ± S.D. *P < 0.05, **P < 0.01, ***P < 0.001.

We performed RT-qPCR to validate the expression of 4 FRGs in HK-2 cells and 7860 cells. As shown in **Figure 14A**, compared with HK-2 cells, the expression levels of four FRGs were significantly increased in 7860 cells (p<0.05), which was consistent with our previous analysis results.

Besides, we also selected CD44 in the 7860 and the Caki-1 cells to further verify the accuracy of our model. The 7860 and the Caki-1 cells were transfected with siRNA-CD44 and siRNA-NC respectively. The results of RT-qPCR showed that the expression level of CD44 in the siRNA-CD44 group was significantly reduced when compared with the blank control group and the siRNA-NC group (**Figure 14B**). Subsequently, we performed a scratch assay to evaluate the effect of knockdown of CD44 on the migration of the 7860 and the Caki-1 cells. Compared with the siRNA-NC group, the migration rate of the siRNA-CD44 group in the 7860 cells was markedly decreased at 6h and 12h. As for the Caki-1 cells, the cell migration rate of the siRNA-CD44 group was markedly decreased at 12h and 24h (**Figure 14C**). Finally, we also performed a proliferation assay. As shown in **Figure 14D**, after transfection of 7860 and Caki-1 cell lines with siRNA-CD44, the cell growth was significantly reduced at 12h, 24h and 48h.

## Discussion

Recently, studies have shown that ferroptosis is involved in a variety of kidney diseases, such as renal ischemia-reperfusion injury (30), acute kidney injury (8), chronic kidney disease (31), etc. CcRCC is the most common tumor in kidney, but its heterogeneity in response to treatment brings difficulties to clinical treatment. Researchers have found that ccRCC is extremely sensitive to ferroptosis, and many studies strongly recommend that targeted activation of ferroptosis can be used as a potential treatment for ccRCC (14, 32). In the past few years, several studies have reported that the regulation of FRGs can affect the process and the prognosis of ccRCC (33, 34). But to our knowledge, there are still not enough studies on the correlation of FRGs with the prognosis and the treatment of ccRCC patients, and most of these studies only focus on a single FRG (18, 19). Therefore, constructing an FRGs-based, accurate stratified model to allow for prognosis and treatment stratification of ccRCC patients will have important clinical significance.

In this study, we systematically explored the expression of 259 FRGs between ccRCC and normal specimens, and finally constructed a novel stratified model for ccRCC patients. The stratified model and a model-based nomogram can accurately predict the prognosis of ccRCC patients in TCGA database. Besides, the model can also provide a reference for the individualized treatment of patients stratified by the model.

Our stratified model contains 4 FRGs (CD44, BID, TRIB3 and TAZ). Among them, BID and TAZ are considered as drivers of ferroptosis, CD44 is considered as a suppressor of ferroptosis and TRIB3 is considered as a marker of ferroptosis. BID is a pro-apoptotic protein and its transactivation to mitochondria can mediate the loss of mitochondrial integrity and dysfunction, as well as the release of pro-apoptotic factors such as endonuclease G, cytochrome C and apoptosis-inducing factor (AIF) and eventually lead to cell apoptosis (35, 36). Neitemeier et al. showed that knocking out the BID gene or using BID inhibitors in neuronal HT-22 cells can prevent mitochondrial damage and cell death induced by erastin (37). Further studies have found that BID can transactivation to mitochondria during the process of ferroptosis, causing mitochondrial damage and cell apoptosis (37). Similarly, research by Jelinek and Wang also found that knocking out the BID gene or using BID inhibitors could protect cells (including normal cells and a variety of cancer cells) from RSL3- or Quercetin-induced cell death by preventing mitochondrial damage (38, 39). The above studies have shown that BID is a key mediator of mitochondrial damage caused by ferroptosis, and ferroptosis may lie upstream of apoptosis. TAZ is a key molecule of the Hippo signaling pathway, which can regulate the density-dependent proliferation of cancer cells (40). Studies have shown that in addition to genetic factors, the sensitivity of many cancers to ferroptosis is also highly affected by non-genetic factors (such as cell density) (41, 42). Yang et al. found that the cell density could affect the sensitivity of ovarian cancer cells to erastin-induced ferroptosis. When plated at low density, ovarian cancer cells are more sensitive to ferroptosis, while plated at high density, the sensitivity is much lower (42). Further studies have found that when the cell density is lower, TAZ becomes dephosphorylated and translocates into the nucleus to drive the expression of ANGPTLA4 and then up-regulates the level of NOX2 to increase the sensitivity of ovarian cancer cells to ferroptosis. Besides, the same group’s research on RCC also obtained similar results. When plated at low density, TAZ can increase the expression of EMP1 and then up-regulate the level of NOX4 to increase the sensitivity of RCC to ferroptosis (32). In our study, the tumor microenvironment analysis showed that the tumor purity of the high-risk group was lower than that of the low-risk group, which indicated the density of ccRCC cells in the high-risk group was lower. This is consistent with the higher TAZ expression in the high-risk group. Combining the higher expression levels of TAZ and BID in this group, we speculate that high-risk ccRCC patients are more sensitive to ferroptosis and targeting ferroptosis to treat such patients may have a better therapeutic effect.

In this study, CD44 was highly expressed in the high-risk group. CD44 is a marker of cancer stem cells (CSCs) (43), which accounts for a relatively small proportion of cancer cells. More and more studies have shown that these cells play a key role in cancer recurrence, invasiveness and drug resistance (43, 44). Ishimoto et al. firstly found that the CD44 variant (CD44v) could stabilize the xCT subunit of the system xc– (cystine-glutamate transporter) to regulate the redox state of gastric cancer cells, therefore promoting tumor growth (45). Recent studies have shown that overexpression of CD44 in cancer cells can promote the interaction between OTUB1 and SLC7A11 to enhance the stability of SLC7A11 (a subunit of glutamate-cystine antiporter system Xc-) and thereby inhibiting the ferroptosis, while the partial depletion of CD44 abolished this interaction (46). Previous studies by Hasegawa et al. also found that the CD44 can form a complex with MUC-1C and xCT to stabilize the xCT protein and inhibit erastin-induced ferroptosis (47). Besides, Müller et al. also found that CD44 could interact with hyaluronates to regulate iron entry into cells and intracellular iron homeostasis (48). The iron entering the cell can further catalyze the oxidative demethylation of histones, thereby promoting the transcriptional expression of the aforementioned MUC-1C and other genes to further inhibit ferroptosis (48). It can be seen that the overexpression of CD44 can enhance the ability of cancer cells to resist oxidative stress through a variety of mechanisms. Combined with the increased sensitivity of the high-risk group to ferroptosis caused by the high expression of BID and TAZ, we speculated that the overexpression of CD44 may play a role in antagonizing TAZ and BID, thereby making cancer cells resistant to lipid peroxidation-mediated ferroptosis. This may require further experiments to prove in the future. Up to now, there are few studies on TRIB3 and ferroptosis. In the experiment of Dixon et al. using erastin to induce endoplasmic reticulum stress and ferroptosis, it was found that after induced by erastin, the expression level of TRIB3 in HT-1080 fibrosarcoma cells increased 2.2 times (17). However, the mechanism causing the increased expression of TRIB3 and its role in ferroptosis is still unclear.

The results of the tumor microenvironment analysis showed that there was a statistical difference between the immune scores of the two groups, which suggested that the abundance and function of immune cells between them were significantly different. Combining TMB and immune infiltration analysis, we found that the high-risk group had higher TMB scores, indicating that such cancer cells were more likely to be detected by the immune system (49). This was consistent with the higher abundance of CD8^+^T cells, activated CD4^+^T cells and plasma cells in the high-risk group. Interestingly, the abundance of Tregs in this group was also found to be significantly higher. As we all know, Tregs can control NK cells, B cells, macrophages and dendritic cells (DCs) through humoral and cell-cell contact mechanisms, thus suppressing tumor immune responses (50). Therefore, we speculated that a large number of Tregs in the tumor microenvironment of high-risk ccRCC can inhibit tumor immune response, thereby helping such cancer cells to escape the immune system. Besides, some studies have also shown that Tregs can promote tumor progression by promoting tumor angiogenesis (51). Through correlation and prognostic analysis, we found that the abundance of Tregs and Mast cells resting was also related to the prognosis of ccRCC. Consistent with our results, a meta-analysis showed that the high abundance of Foxp3^+^Tregs is associated with shorter overall survival (OS) in the majority of solid tumors including renal tumors (52). Besides, in the study of Zhu and Pan, they both found that the higher abundance of mast cells resting is significantly associated with a better prognosis (53, 54). Our TMB analysis was consistent with the previous studies. VHL, PBRM1, TTN, SETD2 and BAP1 are the most frequently mutated genes in ccRCC (49, 55) and the mutation of VHL, TTN, SETD2 and BAP1 in the high-risk group was higher. This is also in line with the ccRCC development model previously proposed by Brugarolas et al. The mutation of VHL is the initial event and the subsequent mutations of PBRM1 and SETD2 may contribute to transformation, while the mutation of BAP1 confers greater aggressiveness (55). Besides, we also found that TMB score combined with risk score can improve the prognostic accuracy for ccRCC patients.

The PD-L1 expression was significantly lower in the high-risk group, which suggested that the PD-L1 antibody may be more beneficial for low-risk patients. On the contrary, the CTLA-4 expression was significantly higher in the high-risk group, indicating that the CTLA-4 antibody would be more effective for high-risk patients. It is known that CTLA-4 is mainly expressed on Tregs cells (56), this is consistent with the higher abundance of Tregs in the tumor microenvironment of high-risk ccRCC. Besides, the sensitivity analysis of traditional anti-tumor drugs showed that Rapamycin, Lapatinib, Erlotinib, AZ628, A-770041, MS-275, Dasatinib and DMOG may be more beneficial to low-risk patients, while 681640, CCT018159, NSC-87887, Lenalidomide and EX-527 may be more sensitive to high-risk patients. Among these drugs, the mTOR inhibitor rapamycin and the double tyrosine kinase inhibitor lapatinib have been shown to inhibit the growth of tumor cells by promoting cell iron death (57, 58).

KEGG pathway enrichment analysis indicated that the targeted genes were mainly enriched in the HIF-1 signaling pathway. It is known that in the case of VHL mutated, HIF-1a is essential for the formation of ccRCC tumors (59). HIF-1a can regulate the expression of many genes (such as Glut1, TGF, VEGF) and promote to establish an angiogenesis favorable microenvironment (60). GSEA indicated that the DNA base repair pathway seems to play a very important role in the high-risk group. Studies have shown that ROS-mediated DNA damage can lead to ferroptosis and apoptosis of cancer cells (61). This seemed to support our finding: during the progress of ccRCC, cancer cells will increase their sensitivity to ferroptosis, so the activated DNA base repair pathway can help them resist the ferroptosis and the subsequent apoptosis due to the imbalance of redox within the cell. Besides, we also found that tight junction pathways were activated in the low-risk group. Previous studies have shown that tight junction can play a barrier role in tumor metastasis. Tumor cells can invade and metastasize only when the tight junction structure is dissociated or changed (62, 63). The tight junction pathway activated in the low-risk group helps to inhibit tumor progression, which is closely related to the better prognosis of such patients. In addition, other signaling pathways that we have identified may also play a potential role in the process of ccRCC, which provides a reference for future research.

In conclusion, we constructed a stratified model for ccRCC patients in this study. The two types of patients stratified by our model differ in many aspects. In terms of prognosis, high-risk patients have shorter OS than lower-risk patients. Early identification of such patients and effective intervention will help improve their long-term survival. In terms of treatment, drug sensitivity analysis suggested that these two types of patients had different sensitivities to traditional anti-tumor drugs. As for ICI, our study showed that high-risk patients might be more sensitive to CLTA-4 antibodies, while low-risk patients might be more beneficial from PD-L1 antibodies. Targeted suppression of Tregs to increase tumor immune response may be effective for high-risk patients. Besides, since high-risk patients seem to be more sensitive to ferroptosis, targeting to induce ferroptosis in the future may also be a better choice for such patients. The stratified model we constructed will help clinicians predict the prognosis of ccRCC and provide a reference for the clinical individualized treatment of ccRCC.

## Data Availability Statement

The original contributions presented in the study are included in the article/**Supplementary Material**. Further inquiries can be directed to the corresponding author.

## Ethics Statement

Our study did not require an ethical board approval because it did not contain human or animal trials.

## Author Contributions

JW, ZS, and QB contributed equally to this work and should be listed as first co-authors. JW, ZS, and QB conceived and designed the manuscript. JW wrote the manuscript. ZS, QB, and WW revised the manuscript. WW supervised the manuscript. All authors have read and approved the final version of this manuscript.

## Conflict of Interest

The authors declare that the research was conducted in the absence of any commercial or financial relationships that could be construed as a potential conflict of interest.

## Publisher’s Note

All claims expressed in this article are solely those of the authors and do not necessarily represent those of their affiliated organizations, or those of the publisher, the editors and the reviewers. Any product that may be evaluated in this article, or claim that may be made by its manufacturer, is not guaranteed or endorsed by the publisher.
