# Supplementary material for: A Ferroptosis-Related Genes Model Allows for Prognosis and Treatment Stratification of Clear Cell Renal Cell Carcinoma: A Bioinformatics Analysis and Experimental Verification
Source: Front Oncol. 2022 Jan 27;12:815223. doi: 10.3389/fonc.2022.815223 (PMC8828561; doi:10.3389/fonc.2022.815223)
Supplement: Supplementary file 3 [file Table_1.docx]

**Baseline characteristics of the ccRCC patients in this study**

| *characteristics* | *Tcga training set* (n=374) | *tcga testing set* (n=156) | *tcga entire set*  (n=530) | |
| --- | --- | --- | --- | --- |
| Overall survival status |  |  | |  |
| Alive | 251 | 106 | | 357 |
| Dead | 123 | 50 | | 173 |
| Follow-up time |  |  | |  |
| Median value (years) | 1304.06 (0-4067) | 1412.55 (2-4537) | | 1336.00 (0-4537) |
| Age |  |  | |  |
| Median value (years) | 60.15 (26-90) | 61.56 (35-85) | | 60.56 (26-90) |
| Gender |  |  | |  |
| Male | 243 | 101 | | 344 |
| female | 131 | 55 | | 186 |
| Histologic grade |  |  | |  |
| G1 | 9 | 5 | | 14 |
| G2 | 159 | 68 | | 227 |
| G3 | 145 | 61 | | 206 |
| G4 | 54 | 21 | | 75 |
| NA | 7 | 1 | | 8 |
| Pathologic stage |  |  | |  |
| Stage I | 188 | 77 | | 265 |
| Stage II | 40 | 17 | | 57 |
| Stage III | 82 | 41 | | 123 |
| Stage IV | 62 | 20 | | 82 |
| NA | 2 | 1 | | 3 |
