# Supplementary material for: A Ferroptosis-Related Genes Model Allows for Prognosis and Treatment Stratification of Clear Cell Renal Cell Carcinoma: A Bioinformatics Analysis and Experimental Verification
Source: Front Oncol. 2022 Jan 27;12:815223. doi: 10.3389/fonc.2022.815223 (PMC8828561; doi:10.3389/fonc.2022.815223)
Supplement: Supplementary file 4 [file Table_2.docx]

**TableS2:** Differentially expressed miRNAs with |log_2_FC| > 1 and P value <0.05.

| **Gene** | **LogFC** | **P Value** |
| --- | --- | --- |
| MT1G | -4.4533101 | 1.42E-28 |
| CD44 | 1.36759436 | 2.19E-18 |
| MTOR | -1.1595562 | 8.30E-37 |
| BLOC1S5-TXNDC5 | -1.8798471 | 2.11E-15 |
| BID | 1.09921094 | 1.10E-35 |
| BNIP3 | 1.57048676 | 5.11E-30 |
| GDF15 | -1.2075655 | 1.94E-16 |
| HMOX1 | 2.36812644 | 1.97E-32 |
| ATP6V1G2 | -1.0651839 | 1.96E-25 |
| NOS2 | 1.01992229 | 0.00242396 |
| TRIB3 | 3.06804198 | 9.85E-36 |
| ALOX12 | 1.27344143 | 2.06E-24 |
| PML | 1.21395759 | 3.33E-36 |
| LURAP1L | 1.04163057 | 1.49E-16 |
| AKR1C1 | -1.1368102 | 1.97E-08 |
| GOT1 | -1.0565477 | 4.68E-28 |
| SLC2A14 | 2.40551348 | 1.76E-19 |
| ALOX15B | 4.27430945 | 2.52E-34 |
| PROM2 | -3.5795551 | 1.99E-39 |
| ALOX5 | 2.08009456 | 3.05E-25 |
| DDIT4 | 2.165568 | 2.24E-31 |
| SCD | 3.0116528 | 3.02E-35 |
| CDO1 | -1.6377847 | 9.38E-34 |
| HILPDA | 4.68684317 | 6.58E-39 |
| SLC2A12 | -3.410524 | 1.12E-39 |
| CA9 | 5.9224971 | 5.35E-38 |
| ATF3 | -1.1927188 | 1.17E-09 |
| PTGS2 | -1.1925186 | 2.04E-14 |
| CBS | -1.0947159 | 3.51E-15 |
| CXCL2 | 1.61573593 | 8.04E-08 |
| EGFR | 1.47185074 | 8.44E-24 |
| NOX4 | -1.2485732 | 0.00341725 |
| ACO1 | -1.0546835 | 1.86E-24 |
| GABARAPL1 | -1.5555595 | 3.00E-37 |
| SLC1A4 | 1.95618222 | 2.03E-38 |
| PSAT1 | -2.4960854 | 4.15E-24 |
| GCH1 | -1.0610052 | 4.83E-20 |
| IFNG | 4.52077265 | 6.90E-30 |
| ALB | -1.3485535 | 5.03E-16 |
| GPT2 | -1.1231596 | 2.93E-18 |
| HAMP | 3.74330925 | 2.84E-29 |
| PLIN2 | 2.979948 | 2.63E-31 |
| MUC1 | -1.5996848 | 2.05E-31 |
| GPX2 | -1.2493006 | 4.76E-28 |
| SLC2A3 | 2.03284323 | 1.51E-26 |
| TFAP2C | -2.228084 | 2.65E-24 |
| NCF2 | 1.76182803 | 8.44E-28 |
| PCK2 | -2.2162223 | 1.11E-09 |
| RRM2 | 2.12392866 | 2.81E-32 |
| TF | 4.13514481 | 1.88E-06 |
| VEGFA | 3.54235369 | 2.71E-37 |
| ENPP2 | 1.59759092 | 4.69E-11 |
| CDKN2A | 4.92264078 | 7.21E-42 |
| SLC7A11 | 1.75974094 | 1.52E-25 |
| ANGPTL7 | -1.0638712 | 2.38E-13 |
| TFR2 | 3.94556162 | 5.32E-29 |
| CAV1 | 2.20654539 | 5.37E-35 |
| MT3 | 3.27686786 | 1.05E-05 |
| TSC22D3 | 1.15127969 | 2.71E-13 |
| MIOX | -2.2186599 | 2.88E-06 |
| TAZ | 1.1000455 | 2.19E-28 |
| CYBB | 1.84737427 | 1.71E-20 |
| PEBP1 | -1.2155456 | 9.00E-33 |
| TNFAIP3 | 1.32093767 | 3.51E-25 |
| DRD4 | 2.01749893 | 1.40E-23 |
| HIF1A | -1.202346 | 6.60E-24 |
| NNMT | 3.87351907 | 9.25E-36 |
| LINC00472 | -1.8580096 | 2.14E-35 |
| AKR1C2 | -1.2670015 | 0.00073359 |
| ACSL4 | -1.5122379 | 2.99E-38 |
| TP63 | -1.1676542 | 1.94E-06 |
| SLC2A1 | 1.97676444 | 6.96E-30 |
| CHAC1 | -2.0237034 | 8.91E-28 |
| ALOX12B | 1.96520949 | 1.93E-16 |
| MYB | 1.39829625 | 1.26E-24 |
| ACSF2 | -3.2315072 | 4.86E-30 |
