# Supplementary material for: A Ferroptosis-Related Genes Model Allows for Prognosis and Treatment Stratification of Clear Cell Renal Cell Carcinoma: A Bioinformatics Analysis and Experimental Verification
Source: Front Oncol. 2022 Jan 27;12:815223. doi: 10.3389/fonc.2022.815223 (PMC8828561; doi:10.3389/fonc.2022.815223)
Supplement: Supplementary file 5 [file Table_3.docx]

TableS3**:** Prognosis-related FRGs with HR ≠ 1 and P < 0.05.

| FRGs | HR | HR.95L | HR.95H | p value |
| --- | --- | --- | --- | --- |
| MT1G | 1.08687432 | 1.00182073 | 1.17914888 | 0.04510034 |
| CD44 | 1.40300008 | 1.18046672 | 1.66748389 | 0.00012162 |
| TLR4 | 0.65600117 | 0.53190276 | 0.80905304 | 8.14E-05 |
| TUBE1 | 1.49850997 | 1.04010979 | 2.1589376 | 0.02992717 |
| HSPB1 | 1.46408679 | 1.20604848 | 1.7773333 | 0.00011626 |
| BID | 3.31199704 | 2.16762441 | 5.06052816 | 3.08E-08 |
| BNIP3 | 0.78426066 | 0.64261305 | 0.95713087 | 0.01679927 |
| BRD4 | 2.22448304 | 1.29270753 | 3.82787651 | 0.00388895 |
| PRKAA2 | 0.44778699 | 0.34335834 | 0.58397645 | 3.03E-09 |
| NOX1 | 3.12456847 | 1.2168449 | 8.02314911 | 0.01789105 |
| TRIB3 | 1.43207692 | 1.23758071 | 1.65713984 | 1.42E-06 |
| ALOXE3 | 2.32852823 | 1.26829968 | 4.27504937 | 0.00639708 |
| ZEB1 | 0.70642154 | 0.55217935 | 0.90374873 | 0.00568916 |
| PML | 1.93306641 | 1.21526625 | 3.07483709 | 0.00538183 |
| LURAP1L | 0.72327456 | 0.57871055 | 0.90395117 | 0.00440588 |
| SNX4 | 0.5261533 | 0.39148546 | 0.7071458 | 2.07E-05 |
| AKR1C1 | 0.75370918 | 0.61900595 | 0.91772548 | 0.00488335 |
| GOT1 | 0.67223719 | 0.53986365 | 0.83706848 | 0.00038595 |
| CDKN1A | 0.78036813 | 0.61889863 | 0.98396473 | 0.03602605 |
| LAMP2 | 0.61562322 | 0.44783015 | 0.84628503 | 0.00280886 |
| LONP1 | 1.89187624 | 1.19672123 | 2.99083498 | 0.00636213 |
| PLIN4 | 1.49356129 | 1.19630204 | 1.86468403 | 0.00039578 |
| PRDX6 | 1.73086905 | 1.10763983 | 2.70476698 | 0.01600378 |
| PHKG2 | 2.83855214 | 1.94057508 | 4.15205694 | 7.59E-08 |
| HERPUD1 | 0.52112501 | 0.38003187 | 0.7146013 | 5.21E-05 |
| EIF2S1 | 0.44414146 | 0.29907571 | 0.6595709 | 5.76E-05 |
| MAP3K5 | 0.62299896 | 0.44939024 | 0.86367631 | 0.00452087 |
| ATG7 | 0.53695918 | 0.29096179 | 0.99093822 | 0.04669211 |
| MAPK1 | 0.59167589 | 0.45514278 | 0.76916603 | 8.83E-05 |
| HIC1 | 1.39355793 | 1.01358256 | 1.91597979 | 0.04105055 |
| SLC7A5 | 1.40400608 | 1.24243297 | 1.58659108 | 5.33E-08 |
| AIFM2 | 1.59262846 | 1.07932518 | 2.35004748 | 0.01905101 |
| DUSP1 | 0.76944484 | 0.65163801 | 0.90854947 | 0.00199417 |
| CHMP5 | 0.42822279 | 0.31676052 | 0.57890662 | 3.52E-08 |
| CBS | 4.55957111 | 1.07130503 | 19.405947 | 0.04005593 |
| CXCL2 | 1.24335521 | 1.09909886 | 1.40654516 | 0.00053678 |
| SIRT1 | 0.52771545 | 0.38618098 | 0.72112199 | 6.02E-05 |
| DPP4 | 0.81996393 | 0.73087127 | 0.91991692 | 0.0007188 |
| TGFBR1 | 0.69139617 | 0.49428438 | 0.96711261 | 0.03114146 |
| DRD5 | 8.86E-05 | 7.08E-08 | 0.11101942 | 0.01034883 |
| LPCAT3 | 0.71376704 | 0.56710064 | 0.89836503 | 0.00406313 |
| PIK3CA | 0.51810253 | 0.3682401 | 0.72895437 | 0.00016018 |
| ACO1 | 0.52342879 | 0.3746405 | 0.73130826 | 0.00014833 |
| FLT3 | 0.50229624 | 0.2983117 | 0.84576472 | 0.00959543 |
| YWHAE | 0.38223819 | 0.22702715 | 0.64356197 | 0.00029681 |
| DDIT3 | 1.52752782 | 1.26635064 | 1.84257121 | 9.50E-06 |
| SLC1A4 | 0.61951409 | 0.44823464 | 0.85624286 | 0.00373252 |
| PSAT1 | 1.26893728 | 1.13381659 | 1.42016076 | 3.38E-05 |
| STEAP3 | 1.39776535 | 1.2415064 | 1.57369142 | 3.09E-08 |
| SLC2A6 | 1.92394527 | 1.46184243 | 2.53212339 | 3.02E-06 |
| BECN1 | 0.67781104 | 0.48728331 | 0.94283511 | 0.02091313 |
| CISD2 | 0.61371403 | 0.4239918 | 0.88833065 | 0.00966679 |
| HAMP | 1.72467654 | 1.31424376 | 2.26328575 | 8.47E-05 |
| PLIN2 | 0.86447675 | 0.77831395 | 0.96017815 | 0.00655713 |
| ULK1 | 1.77870793 | 1.34038686 | 2.36036474 | 6.62E-05 |
| EPAS1 | 0.6677558 | 0.57335811 | 0.77769514 | 2.07E-07 |
| SCP2 | 0.55695316 | 0.43911606 | 0.70641193 | 1.40E-06 |
| SLC40A1 | 0.62259032 | 0.52460214 | 0.73888129 | 5.85E-08 |
| HRAS | 1.39052961 | 1.04444066 | 1.85129962 | 0.02396238 |
| JDP2 | 0.71412596 | 0.52727892 | 0.96718429 | 0.0295882 |
| HSD17B11 | 0.70400526 | 0.54755106 | 0.90516382 | 0.00620046 |
| RRM2 | 1.49360027 | 1.19808594 | 1.86200479 | 0.00036159 |
| G6PD | 2.04013309 | 1.5697023 | 2.65154929 | 9.75E-08 |
| ENPP2 | 0.83089563 | 0.73604993 | 0.93796293 | 0.00273905 |
| CDKN2A | 1.73366876 | 1.37041747 | 2.19320567 | 4.50E-06 |
| SLC7A11 | 2.00692532 | 1.31649798 | 3.05944202 | 0.00120286 |
| AGPAT3 | 0.63284187 | 0.46247047 | 0.8659771 | 0.00424719 |
| MAPK8 | 0.42917946 | 0.26411014 | 0.6974174 | 0.00063839 |
| HELLS | 1.71459415 | 1.09542362 | 2.68374084 | 0.01834066 |
| MAPK3 | 0.67672311 | 0.4604243 | 0.9946351 | 0.04688461 |
| TSC22D3 | 0.83628737 | 0.70635147 | 0.99012545 | 0.03797279 |
| MIOX | 0.86799036 | 0.80028209 | 0.94142712 | 0.00063415 |
| GABARAPL2 | 0.51943781 | 0.30862747 | 0.87424375 | 0.01366585 |
| TAZ | 2.35640468 | 1.70154323 | 3.26329824 | 2.48E-07 |
| MTDH | 0.69761567 | 0.5094155 | 0.95534515 | 0.02478454 |
| ASNS | 2.07244739 | 1.64418114 | 2.6122658 | 6.83E-10 |
| KRAS | 0.57098521 | 0.39613418 | 0.82301432 | 0.00266324 |
| PEBP1 | 0.68783815 | 0.51712843 | 0.91490099 | 0.01013925 |
| ATF4 | 1.60610178 | 1.13971286 | 2.26334457 | 0.00678594 |
| NCOA4 | 0.53905056 | 0.44043361 | 0.65974871 | 2.04E-09 |
| HNF4A | 0.82849274 | 0.72873165 | 0.94191081 | 0.00405106 |
| ALOX15 | 5.87750525 | 1.43753211 | 24.0308149 | 0.01369776 |
| DRD4 | 1.90086861 | 1.28545385 | 2.81091498 | 0.00129056 |
| ANO6 | 0.77617092 | 0.60604359 | 0.99405604 | 0.04472943 |
| TXNIP | 0.74625636 | 0.59577448 | 0.93474726 | 0.01085816 |
| SLC1A5 | 1.79641973 | 1.38842664 | 2.32430274 | 8.33E-06 |
| SOCS1 | 1.48752386 | 1.26211505 | 1.75318982 | 2.17E-06 |
| GCLC | 0.57095844 | 0.3973005 | 0.82052134 | 0.00245245 |
| EGLN2 | 1.52128125 | 1.16666215 | 1.98369051 | 0.00194632 |
| PRKAA1 | 0.60459544 | 0.43621588 | 0.8379696 | 0.00251626 |
| IL6 | 1.20638557 | 1.09797227 | 1.32550354 | 9.41E-05 |
| RB1 | 0.64273994 | 0.50818173 | 0.81292695 | 0.00022595 |
| NRAS | 0.59912709 | 0.44480054 | 0.80699828 | 0.00074888 |
| AURKA | 1.85561912 | 1.39021733 | 2.47682304 | 2.71E-05 |
| MAPK9 | 0.57212921 | 0.35892655 | 0.91197443 | 0.01890966 |
| VDAC2 | 0.60887282 | 0.39076136 | 0.94872766 | 0.02833901 |
| HSF1 | 2.40058735 | 1.57409223 | 3.66104319 | 4.76E-05 |
| SAT1 | 1.51904691 | 1.14797222 | 2.01006913 | 0.0034376 |
| CHAC1 | 1.62840115 | 1.34152297 | 1.97662684 | 8.16E-07 |
| IREB2 | 0.53340184 | 0.38989415 | 0.72973016 | 8.48E-05 |
| SRC | 2.8567663 | 1.97343246 | 4.13549177 | 2.67E-08 |
| FANCD2 | 2.08036749 | 1.29050006 | 3.35368359 | 0.00264069 |
| KLHL24 | 0.66565139 | 0.46431948 | 0.95428211 | 0.0267875 |
| NFE2L2 | 0.69215479 | 0.5074088 | 0.94416623 | 0.02019891 |
| FTL | 1.38332066 | 1.09590816 | 1.74610986 | 0.00632057 |
