# Supplementary material for: A Ferroptosis-Related Genes Model Allows for Prognosis and Treatment Stratification of Clear Cell Renal Cell Carcinoma: A Bioinformatics Analysis and Experimental Verification
Source: Front Oncol. 2022 Jan 27;12:815223. doi: 10.3389/fonc.2022.815223 (PMC8828561; doi:10.3389/fonc.2022.815223)
Supplement: Supplementary file 6 [file Table_4.docx]

**TableS4:** The HR and p values of 16 OC-FRGs.

| FRGs | HR | HR.95L | HR.95H | p value |
| --- | --- | --- | --- | --- |
| CD44 | 1.40300008 | 1.18046672 | 1.66748389 | 0.00012162 |
| HAMP | 1.72467654 | 1.31424376 | 2.26328575 | 8.47E-05 |
| BID | 3.31199704 | 2.16762441 | 5.06052816 | 3.08E-08 |
| DRD4 | 1.90086861 | 1.28545385 | 2.81091498 | 0.00129056 |
| TRIB3 | 1.43207692 | 1.23758071 | 1.65713984 | 1.42E-06 |
| RRM2 | 1.49360027 | 1.19808594 | 1.86200479 | 0.00036159 |
| CDKN2A | 1.73366876 | 1.37041747 | 2.19320567 | 4.50E-06 |
| SLC7A11 | 2.00692532 | 1.31649798 | 3.05944202 | 0.00120286 |
| CXCL2 | 1.24335521 | 1.09909886 | 1.40654516 | 0.00053678 |
| PML | 1.93306641 | 1.21526625 | 3.07483709 | 0.00538183 |
| TAZ | 2.35640468 | 1.70154323 | 3.26329824 | 2.48E-07 |
| PEBP1 | 0.68783815 | 0.51712843 | 0.91490099 | 0.01013925 |
| AKR1C1 | 0.75370918 | 0.61900595 | 0.91772548 | 0.00488335 |
| GOT1 | 0.67223719 | 0.53986365 | 0.83706848 | 0.00038595 |
| ACO1 | 0.52342879 | 0.3746405 | 0.73130826 | 0.00014833 |
| MIOX | 0.86799036 | 0.80028209 | 0.94142712 | 0.00063415 |
