# Supplementary material for: A Ferroptosis-Related Genes Model Allows for Prognosis and Treatment Stratification of Clear Cell Renal Cell Carcinoma: A Bioinformatics Analysis and Experimental Verification
Source: Front Oncol. 2022 Jan 27;12:815223. doi: 10.3389/fonc.2022.815223 (PMC8828561; doi:10.3389/fonc.2022.815223)
Supplement: Supplementary file 7 [file Table_5.docx]

**TableS5:** Gene ontology (GO) enrichment analysis of differentially expressed FRGs.

| Ontology | Term | P value | Count |
| --- | --- | --- | --- |
| BP | response to hypoxia | 3.90E-14 | 17 |
| BP | response to decreased oxygen levels | 6.39E-14 | 17 |
| BP | response to oxygen levels | 1.77E-13 | 17 |
| BP | reactive oxygen species metabolic process | 3.05E-13 | 15 |
| BP | cellular response to oxidative stress | 1.19E-11 | 14 |
| BP | response to oxidative stress | 1.92E-11 | 16 |
| BP | carboxylic acid biosynthetic process | 2.75E-11 | 16 |
| BP | organic acid biosynthetic process | 2.84E-11 | 16 |
| BP | intrinsic apoptotic signaling pathway | 1.01E-10 | 13 |
| BP | response to metal ion | 1.41E-10 | 14 |
| CC | NADPH oxidase complex | 2.15E-05 | 3 |
| CC | apical part of cell | 8.57E-05 | 8 |
| CC | basolateral plasma membrane | 0.00015275 | 6 |
| CC | apical plasma membrane | 0.00017406 | 7 |
| CC | caveola | 0.0002211 | 4 |
| CC | lipid droplet | 0.00023194 | 4 |
| CC | rough endoplasmic reticulum | 0.00027914 | 4 |
| CC | mitochondrial outer membrane | 0.00051842 | 5 |
| CC | plasma membrane raft | 0.0007174 | 4 |
| CC | oxidoreductase complex | 0.00079425 | 4 |
| MF | oxidoreductase activity, acting on single donors with incorporation of molecular oxygen | 1.51E-11 | 7 |
| MF | oxidoreductase activity, acting on single donors with incorporation of molecular oxygen, incorporation of two atoms of oxygen | 1.01E-09 | 6 |
| MF | iron ion binding | 1.08E-08 | 9 |
| MF | oxidoreductase activity, acting on NAD(P)H | 2.58E-07 | 7 |
| MF | dioxygenase activity | 1.86E-06 | 6 |
| MF | ferric iron binding | 1.04E-05 | 3 |
| MF | superoxide-generating NADPH oxidase activity | 1.38E-05 | 3 |
| MF | carbohydrate transmembrane transporter activity | 1.50E-05 | 4 |
| MF | heme binding | 1.82E-05 | 6 |
| MF | coenzyme binding | 2.33E-05 | 8 |
