# Supplementary material for: A Ferroptosis-Related Genes Model Allows for Prognosis and Treatment Stratification of Clear Cell Renal Cell Carcinoma: A Bioinformatics Analysis and Experimental Verification
Source: Front Oncol. 2022 Jan 27;12:815223. doi: 10.3389/fonc.2022.815223 (PMC8828561; doi:10.3389/fonc.2022.815223)
Supplement: Supplementary file 8 [file Table_6.docx]

**TableS6:** Kyoto Encyclopedia of Genes and Genomes (KEGG) pathways analysis of differentially expressed FRGs.

| Id | Term | P value | Count |
| --- | --- | --- | --- |
| hsa04066 | HIF-1 signaling pathway | 3.89E-09 | 10 |
| hsa00590 | Arachidonic acid metabolism | 4.23E-06 | 6 |
| hsa04216 | Ferroptosis | 9.71E-06 | 5 |
| hsa01230 | Biosynthesis of amino acids | 0.000185734 | 5 |
| hsa05140 | Leishmaniasis | 0.000210317 | 5 |
| hsa01210 | 2-Oxocarboxylic acid metabolism | 0.000311016 | 3 |
| hsa05206 | MicroRNAs in cancer | 0.000318812 | 9 |
| hsa05167 | Kaposi sarcoma-associated herpesvirus infection | 0.000419809 | 7 |
| hsa00270 | Cysteine and methionine metabolism | 0.000426133 | 4 |
| hsa00220 | Arginine biosynthesis | 0.000486804 | 3 |
